# Late pharmacologic conditioning with volatile anesthetics after cardiac surgery

**DOI:** 10.1186/cc11676

**Published:** 2012-10-14

**Authors:** Marc P Steurer, Martina A Steurer, Werner Baulig, Tobias Piegeler, Martin Schläpfer, Donat R Spahn, Volkmar Falk, Pamela Dreessen, Oliver M Theusinger, Edith R Schmid, David Schwartz, Thomas A Neff, Beatrice Beck-Schimmer

**Affiliations:** 1 Institute of Anaesthesiology, University Hospital Zurich, Raemistrasse 100, CH-8091 Zurich, Switzerland; 2 Institute of Physiology and Zurich Center for Integrative Human Physiology, University of Zurich, Winterthurerstrasse 190, CH-8057 Zurich, Switzerland; 3 Department of Anesthesiology and Perioperative Care, University of California at San Francisco, San Francisco General Hospital, 1001 Potrero Ave, San Francisco, CA 94110, USA; 4 Pediatric Critical Care, Department of Pediatrics, University of California at San Francisco, 505 Parnassus Ave, San Francisco, CA 94143, USA; 5 Department of Cardiac Surgery, University Hospital Zurich, Raemistrasse 100, CH-8091 Zurich, Switzerland; 6 Division of Cardiac Anaesthesia, Institute of Anaesthesiology, University Hospital Zurich, Raemistrasse 100, CH-8091 Zurich, Switzerland; 7 Department of Anesthesiology, University of Illinois at Chicago, 1740 West Taylor Street, Suite 3200W, MC 515, Chicago, IL 60612, USA; 8 Department of Anaesthesiology, Intensive Care and Emergency Medicine, Cantonal Hospital, Postfach, CH-8596 Muensterlingen, Switzerland

## Abstract

**Introduction:**

The aim of this randomized controlled trial was to investigate whether volatile anesthetics used for postoperative sedation have any beneficial effects on myocardial injury in cardiac surgery patients after on-pump valve replacement.

**Methods:**

Anesthesia was performed with propofol. After arrival in the intensive care unit (ICU), 117 patients were randomized to be sedated for at least 4 hours with either propofol or sevoflurane. Sevoflurane was administered by using the anesthetic-conserving device. Troponin T, creatine kinase, creatine kinase from heart muscle tissue, myoglobin, and oxygenation index were determined on arrival at the ICU, 4 hours after sedation, and in the morning of the first postoperative day (POD1). Primary end points were cardiac injury markers on POD1. As secondary end points oxygenation, postoperative pulmonary complications, and ICU and hospital stay were documented.

**Results:**

Fifty-six patients were analyzed in the propofol arm, and 46 patients in the sevoflurane arm. Treatment groups were comparable with regard to patient demographics and intraoperative characteristics. Concentration of troponin T as the most sensitive marker for myocardial injury at POD1 was significantly lower in the sevoflurane group compared with the propofol group (unadjusted difference, -0.4; 95% CI, -0.7 to -0.1; *P *< 0.01; adjusted difference, -0.2; 95% CI, -0.4 to -0.02; *P *= 0.03, respectively).

**Conclusions:**

The data presented in this investigation indicate that late postconditioning with the volatile anesthetic sevoflurane might mediate cardiac protection, even with a late, brief, and low-dose application.

**Trial registration:**

ClinicalTrials.gov: NCT00924222.

## Introduction

Cardiac surgery requiring extracorporeal circulation (ECC) is a common procedure, which is used for valve repair, valve replacement, as well as for coronary artery bypass grafting. During ECC, the myocardium is exposed to transient ischemia, followed by reperfusion, sometimes leading to myocardial infarction. This is linked to an increased long-term incidence of adverse cardiovascular events [1]. Additionally, a systemic inflammatory response of various degrees is observed with cardiopulmonary bypass, possibly leading to systemic inflammatory response syndrome and/or single or multiple organ dysfunctions [2-5]. Both ischemia-reperfusion injury and the inflammatory response syndrome negatively affect patient outcome.

For several years, inhalational agents have been successfully used for sedation of ventilator-dependent patients in the intensive care unit (ICU) and have become a valuable alternative to commonly used intravenous drugs [6]. Studies have shown that the method of sedation with volatile anesthetics [7] results in shorter time to extubation and faster mental recovery compared with intravenously administered sedatives [8,9]. Recently, a new tool for postoperative volatile sedation of ventilated patients in the ICU has been established: the Anaesthetic Conserving Device (AnaConDa; Sedana Medical, Uppsala, Sweden).

The protective effects of volatile anesthetics on organ preservation have been studied extensively. Several clinical trials have demonstrated that volatile anesthetics decrease cardiac injury after procedures involving the use of ECC [10-13]. Most often, volatile anesthetics have been used in a preconditioning manner or during the entire surgical procedure. This randomized controlled trial, in contrast, examined the effect of sevoflurane in a clinical setting of late postconditioning in patients undergoing cardiac surgery with ECC. Sevoflurane was therefore administered only postoperatively by using the AnaConDa system [14,15]. We hypothesized that late and short-time application of sevoflurane in low anesthetic gas concentration would attenuate myocardial ischemia-reperfusion injury after cardiopulmonary bypass procedures and possibly have a positive impact on pulmonary function.

## Materials and methods

### Study design

This is a prospective randomized parallel group trial comparing postoperative propofol with sevoflurane sedation in the ICU after on-pump cardiac surgery. All patients between 18 and 90 years of age, scheduled for elective cardiac surgery requiring the use of ECC at the University Hospital Zurich, Switzerland, were eligible. Exclusion criteria were poor cardiac baseline function, defined as an ejection fraction of < 30%, significant coronary impairment (CCS ≥ 3 or myocardial infarction within 7 days before the surgery date), emergency procedures, previous cardiac surgery, chronic pulmonary disease (FEV_1 _< 80% or FEV_1_/FVC < 70% of predicted value), renal dysfunction (creatinine clearance, < 60 ml/min), insulin-dependent diabetes mellitus, pregnancy, and current steroid treatment.

Randomization was computer generated with prestratification for the following surgery groups: (a) aortic valve surgery, (b) mitral valve surgery, and (c) combined procedures with coronary artery bypass grafting (CABG) or replacement of the ascending aorta. The envelope was opened by an investigator at the end of the case. Anesthesiologists and surgeons were blinded to the intervention.

The local ethics committee (Ethic committee, Kantonale Ethikkommission, University Hospital Zurich, Switzerland) approved the trial (StV 5-2007). The study was registered in ClinicalTrials.gov as NCT00924222. Written informed consent was obtained from all study subjects.

### Anesthetic management

Preoperatively patients underwent routine clinical and laboratory examinations. Before surgery, they received oral midazolam at the clinical discretion of the anesthesiologist. For the surgical procedure, patients were monitored in a standard fashion with a five-lead electrocardiogram, pulse oximetry, invasive blood pressure measurement, central venous pressure measurement, bispectral index (BIS), and transesophageal echocardiography. Direct measurement of pulmonary artery pressure was used in more complex cases. The cardiopulmonary bypass (CPB) technique was nonpulsatile, using a roller pump. Active cooling was performed, aiming at temperatures of 32°C to 34°C. Crystalloid cardioplegia was used without hot shot. All patients received general anesthesia with a target-controlled infusion of propofol, fentanyl boluses, plus remifentanil as a continuous infusion. Muscular relaxation was achieved with pancuronium at the induction of anesthesia. Volatile anesthetics were not applied to the patient in the operating room at all. After surgery, patients were transferred to the ICU under continuous analgosedation with propofol and remifentanil.

### ICU management

Patients in the control group received propofol titrated by the critical care team within a range of 0.5 to 4.0 mg/kg BW per hour to achieve continuous sedation (total intravenous application, no target-controlled infusion). Propofol was started at a rate of 2 mg/kg/h and adjusted according to the sedation score and hemodynamics. Remifentanil at a dose of 0.05 to 0.2 μg/kg BW per minute was added as needed to achieve analgesia. In the treatment group, patients were switched to an inhalational sedation regimen with sevoflurane immediately after arrival at the ICU. For this purpose, sevoflurane (Sevorane; Abbot, Abbot Park, IL, USA) was applied via the AnaConDa system at a starting dose of an age-adjusted minimum alveolar concentration (MAC) of 0.5 [16], and was titrated to balance sedation. The end-tidal concentration of sevoflurane was measured by using a Dräger Scio gas module (Dräger Medical, Lübeck, Germany). Remifentanil was applied to patients in the sevoflurane arm at the same does as in the propofol group (0.05 to 0.2 μg/kg/BW). The minimal duration of sedation was 4 hours. During sedation, the patients were monitored with five-lead electrocardiogram, pulse oximetry, invasive blood pressure measurement, and central venous pressure measurement. The AnaConDa module was incorporated in the respiratory circuit of all patients in both groups to control for any unknown effects of the system itself. A CONTRAfluran active charcoal filter (ZeoSys GmbH, Berlin, Germany) was used on the expiratory valve of the ventilator to minimize environmental contamination with sevoflurane. For mechanical ventilation, the ventilator Evita XL (Dräger, Lübeck, Germany) was used in biphasic positive airway pressure (BiPAP) mode with a first positive endexpiratory pressure (PEEP) of 6 cm H_2_O and tidal volumes of 6 to 9 ml/kg. The settings were adjusted to maintain a p_a_CO_2 _of 5.0 to 6.0 kPa. The F_i_O_2 _was set to reach an age-adapted p_a_O_2 _between 8.5 and 10 kPa. Once the patient fulfilled extubation criteria (adequate cardiac function, hemodynamic stability, no coagulopathy, no bleeding, adequate pulmonary function and respiratory effort, including normal postoperative chest radiograph and sufficient blood gas analysis, as judged by the intensivist), the application of propofol or sevoflurane was stopped.

Any postoperative nausea and vomiting (PONV) was treated according to the following scheme: (a) topisetron, 2 mg, plus droperidol, 0.5 mg, intravenously (iv) applied; (b) repeated droperidol, 0.5 mg, iv; (c) meclozin/pyridoxin/caffeine (50/50/20 mg) suppository; and (d) metoclopramide, 10 mg iv. Steroids and propofol were not used for this purpose.

### Primary and secondary outcomes

The primary outcome was defined as cardiac injury on the first postoperative day (POD1) measured by troponin T 12 to 18 hours after surgery. Additional biochemical outcomes were creatine kinase (CK), myocardium-specific creatine kinase (CK-MB), and myoglobin. All laboratory values were determined on arrival in the ICU (considered baseline values), 4 hours after initiating postoperative sedation in the ICU, and in the morning of POD1. The following normal ranges are accepted for the different parameters: troponin, < 0.014 μg/L; CK, < 190 U/L; CK-MB, 28 to 72 U/L; and myoglobin, < 24 μg/L.

Secondary end points included oxygenation index (p_a_O_2_/F_i_O_2_) after 4 hours of sedation before extubation and at POD1, incidence of postoperative pulmonary complications (any of the following: temperature > 38.5°C plus productive cough, radiologic signs of pneumonia or pathologic organisms in Gram stain or culture; initiation of antimicrobial therapy; need for reintubation) during hospitalization, duration of ICU and hospital stay, and the need for antiemetics.

To control for possible remaining confounders, the following parameters were additionally recorded: ECC time, aortic cross-clamp (ACC) time, and administration of blood products.

### Statistics

The study was powered to detect a difference of 0.3 U/L in troponin on POD1 between the two groups with a standard deviation of 0.5 U/L, a β of 0.8, and an α of 0.05. The expected number in each group was 44.

The data analyst was masked for group assignment when performing the statistical analyses, and the randomization code was broken only after the analyses were completed. A per-protocol analysis was performed: we analyzed all patients according to the randomization and whether they received the randomly assigned intervention. We did not include patients in the analyses who were extubated early and who could not be sedated appropriately, because we did not collect outcome data for these patients.

For all continuous outcomes, unadjusted and adjusted linear regression models were performed. For all dichotomous outcomes, unadjusted and adjusted logistic regression models were calculated. The analyses were adjusted for potential confounders. The adjusted models included patient age, need for blood products during the case, as well as the duration of ECC and ACC. For the cardiac injury markers, the models also included baseline values of the corresponding cardiac injury marker on admission to the ICU. For the linear regression models, the distributions of the residuals were evaluated (normal distribution). Statistical significance was defined as *P *< 0.05. STATA was used for all analyses (STATA for Mac, version 12.0; Stata Corp, College Station, TX, USA).

## Results

**Figure 1:**
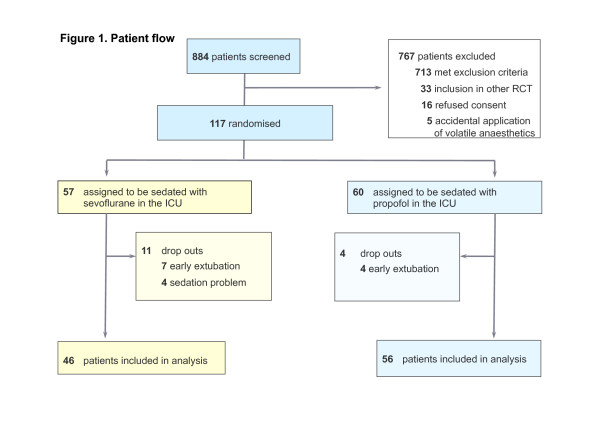
**Enrollment and randomization of patients**. ICU, intensive care unit; OR, operating room; RCT, randomized controlled trial.

Between October 2007 and September 2009, 884 patients were assessed for eligibility (Figure 1). Seven hundred sixty-seven patients did not qualify for the study, 16 refused consent, 33 were included in other RCTs, 713 met exclusion criteria, and five patients had received a volatile anesthetic during surgery. One hundred seventeen consented and were randomized, 57 patients for sevoflurane and 60 for propofol sedation. Eleven and 4 dropouts were found in the sevoflurane and propofol groups, respectively: in the sevoflurane group, 7 patients were extubated earlier than the foreseen 4 hours, and in 4 patients, the AnaConDa device had to be removed because of sedation problems, and sedation was continued with propofol. In the propofol group, 4 early extubations were observed. The primary analysis was performed with 46 patients randomized to sevoflurane, 56 patients to propofol.

The baseline characteristics and intraoperative parameters are presented in Table 1. As expected with stratified randomization, the distribution of the types of surgery was even between both groups: 28 patients in the propofol group and 27 patients in the sevoflurane group received aortic valve surgery. Nine patients in the propofol group and 4 patients in the sevoflurane group had a mitral valve procedure. Nineteen patients in the propofol group and 15 in the sevoflurane group had combined cardiac surgery on either the aortic or the mitral valve, together with CABG surgery or replacement of the ascending aorta.

**Table 1:** Baseline characteristics and intraoperative parameters

	Sevoflurane group (*n *= 46)	Propofol group (*n *= 56)
Age in years (mean, SD)	63 (12.4)	64 (14.7)
Male (%)	69	67
Body mass index in kg/m^2 ^(mean, SD)	26.6 (3.7)	27.1 (3.8)
ECC time (minutes; mean, SD)	141 (39)	152 (49)
ACC time (minutes; mean, SD)	92 (31)	101 (34)
Crystalloids (milliliters; mean, SD)	1,390 (450)	1,480 (610)
Colloids (milliliters; mean, SD)	1,490 (740)	1,420 (760)
Blood products in units (median, IQR)	0 (1)	0 (2)
Troponin T baseline (μg/L; mean, SD)	0.65 (0.6)	0.87 (0.9)
CK baseline (U/L; mean, SD)	373 (191)	493 (337)
CK-MB baseline (U/L; mean, SD)	47.5 (56.4)	50.6 (31.1)
Myoglobin baseline (μg/L; mean, SD)	359 (149)	477 (271)

ACC, aortic cross clamp; CK, creatine kinase; CK-MB, creatine kinase, heart muscle tissue; ECC, extracorporeal circulation. Baseline refers to the time-point at the end of the case before starting the sedation with either sevoflurane or propofol.

Primary outcome, troponin T on POD1, reached statistical significance in the unadjusted as well as the linear regression model, adjusted for age of the patient, ECC, blood products, as well as the baseline troponin level. On POD1, the mean of troponin in the sevoflurane group was 0.4 μg/L lower than that in the propofol group (95% CI, -0.7 to -0.1; *P *< 0.01). In the adjusted model, this difference reached a value of 0.2 U/L (CI, -0.4 to -0.02; *P *= 0.03) (Table 2). Aortic cross-clamp time was dropped from the multiple regression model because of its nonsignificant impact on the results as well as because of concerns for collinearity with the ECC time. In the linear regression model, values for CK reached significance on POD1 in the unadjusted (difference, -258; CI, -434 to -83; *P *< 0.01), as well as in the adjusted model (without adjustment for ACC) (difference, 169; CI, -331 to -8; *P *= 0.04) (Table 2).

**Table 2:** Linear regression analysis to compare cardiac markers between the sevoflurane and propofol groups (*n *= 102)

Cardiac marker (U/L)	Unadjusted difference in means (point estimate)	95% Cl	Adjusted difference in means (point estimate)	95% CI
Troponin T, 4 hours (μg/L)	-0.3	-0.7, 0.1	-0.1	-0.2, 0.1
CK, 4 hours (U/L)	**-140^a^**	**-250, -30**	-38	-96, 20
CK-MB, 4 hours (U/L)	-2.4	-23.9, 19.2	1.2	-6.4, 8.7
Myoglobin, 4 hours (μg/L)	**-113^a^**	**-187, -39**	-42	-100, 16
Troponin T, POD1 (μg/L)	**-0.4^a^**	**-0.7, -0.1**	**-0.2^a^**	**-0.4, -0.02**
CK, POD1 (U/L)	**-258^a^**	**-434, -83**	**-169^a^**	**-331, -8**
CK-MB, POD1 (U/L)	-4.6	-27.5, 18.3	-1.1	-13.2, 11.0
Myoglobin, POD1 (μg/L)	-107	-217, 3	-48	-157, 60

The model is adjusted for age, blood products, baseline value of corresponding cardiac injury marker on ICU admission, and extracorporeal circulation (ECC) time. The propofol group is the reference. ^a^*P *< 0.05, statistically significant.

4 hours, 4 hours after ICU admission; CK, creatine kinase; CK-MB, creatine kinase, heart muscle tissue; POD1, first postoperative day.

In the subgroup of patients with aortic valve surgery (28 in the propofol and 27 in the sevoflurane group), the following results were assessed: The unadjusted difference in means between the propofol group and the sevoflurane group was 0.5 μg/L for troponin T on POD1 (CI, -0.8 to -0.1; *P *= 0.006) (Table 3). The adjusted difference in means between the groups was 0.3 μg/L (CI, -0.6 to -0.1; *P *= 0.02). The same model of multiple linear regression was used as described earlier.

**Table 3:** Linear regression analysis to compare cardiac markers between sevoflurane and propofol for the subgroup with aortic valve surgery (*n *= 55)

Cardiac marker (U/L)	Unadjusted difference in means (point estimate)	95% Cl	Adjusted difference in means (point estimate)	95% CI
Troponin T, 4 hours (μg/L)	-0.5	-1.0, 0.0	-0.2	-0.4, 0.1
CK, 4 hours (U/L)	**-146^a^**	**-284, -8**	-46	-146, 54
CK-MB, 4 hours (U/L)	-10.3	-25.1, 4.6	-1.3	-10.5, 7.7
Myoglobin, 4 hours (μg/L)	-60	-146, 27	4	-51, 58
Troponin T, POD1 (μg/L)	**-0.5^a^**	**-0.8, -0.1**	**-0.3^a^**	**-0.6, -0.1**
CK, POD1 (U/L)	**-250^a^**	**-497, -2**	-120	-352, 112
CK-MB, POD1 (U/L)	-9.7	-21.7, 2.4	-4.8	-15.0, 5.4
Myoglobin, POD1 (μg/L)	-36	-141, 69	33	-47, 112

4 hours, 4 hours after ICU admission; creatine kinase; CK-MB, creatine kinase, heart muscle tissue;

POD1, first postoperative day. The model is adjusted for age, blood products, baseline value of corresponding cardiac injury marker on ICU admission, and extracorporeal circulation (ECC) time. The propofol group is the reference. **^a^***P *< 0.05, statistically significant.

For CK values, the difference in mean value was 250 U/L at POD1 in the unadjusted model (CI, -497 to -2; *P *= 0.048); after adjustment, no significant result was obtained.

The mean oxygenation index in the sevoflurane group was 42 points higher on POD1 compared with the propofol group in the unadjusted model (*P *= 0.04) (Table 4). After adjusting for age, blood products, and ECC time, the difference decreased to 27 points and was no longer significant. Postoperative pulmonary complications were lower in the sevoflurane group, with an OR of 0.4, but not statistically significant in comparison with the propofol group I in either the unadjusted logistic regression model or after adjusting for age, blood products, and ECC time (Table 5).

**Table 4:** Linear regression analysis to compare secondary outcomes at different times between the sevoflurane and propofol groups (*n *= 102)

Oxygenation index (mm Hg)	Unadjusted difference in means (point estimate)	95% Cl	Adjusted difference in means (point estimate)	95% CI
p_a_O_2_/F_i_O_2_, 4 hours (mm Hg)	21	-5, 48	12	-13, 38
p_a_O_2_/F_i_O_2_, POD1 (mm Hg)	**42**^a^	**2, 81**	27	-9, 64

ICU stay (days)	-0.005	-0.6, 0.6	0.07	-0.5, 0.7
Hospital stay (days)	-0.4	-1.9, 1.2	-0.2	-1.7, 1.4
Creatinine, POD1 (μ*M*)	-3.8	-11.8, 4.1	-1.8	-9.6, 6.0

4 hours, 4 hours after ICU admission; ICU, intensive care unit; POD1, first postoperative day. The model is adjusted for age, blood products, and ECC time. The propofol group is the reference. ^a^*P *< 0.05, statistically significant.

**Table 5:** Logistic regression analysis to compare secondary binary outcomes between the sevoflurane and propofol groups (*n *= 102)

	Unadjusted OR	95% Cl	Adjusted OR	95% CI
Postoperative pulmonary complications	0.4	0.2, 1.1	0.4	0.2, 1.2
PONV	1.4	0.6, 3.0	1.3	0.6, 2.8

PONV, postoperative nausea and vomiting. The model is adjusted for age, blood products, and extracorporeal circulation (ECC) time. The propofol group is the reference.

The length in ICU stay and hospitalization time did not differ between the two groups in the adjusted and in the unadjusted model (Table 4).

Plasma creatinine levels on POD1 as well as the incidence of PONV and postoperative pulmonary complications were comparable between both groups, and no statistical significant differences were found in the adjusted and in the unadjusted models (Tables 4 and 5).

## Discussion

The main result of this randomized controlled trial is that patients after aortic and mitral valve replacement in combination with CABG or ascending aorta surgery receiving a 4-hour sedation with the volatile anesthetic had lower troponin T values as the most-specific cardiac injury marker at POD1 compared with propofol sedation.

It is well known from experimental observations that halogenated anesthetics exert many non-anesthetic properties, causing an endogenous adaptive response of cells to injury, such as that of myocardiocytes to an ischemic insult [17,18]. In open-heart surgery, myocardial ischemia and reperfusion is an inherent and inevitable part of the surgical procedure itself. Presence and duration of ACC and ECC time are thereby major factors determining the extent of the inflammatory response, injury, and finally, impairing postoperative organ function.

Relevant protection of postoperative cardiac function was first described in 1999 in a small clinical study that used a preconditioning protocol with isoflurane before cross-clamping [10]. The study revealed less release of CK-MB and troponin, indicating attenuated cardiac injury in the isoflurane group. The cardioprotective potential of various volatile anesthetics, applied during surgery, measured by a reduction in biomarkers of cardiac injury, has been confirmed by many authors [11-13,19,20]. A meta-analysis performed in 2007 clearly suggested that sevoflurane or desflurane is able to reduce the incidence of myocardial infarction and postoperative mortality after cardiac surgery [21]. Some of these studies even showed benefits with regard to mechanical ventilation and/or length of hospital stay [12,13]. Different windows of pharmacologic protection with early and late pharmacologic preconditioning by the use of volatile anesthetics have been described in the literature, both probably involving different signaling pathways, which are still not understood in detail. Reactive oxygen species, apoptotic signaling pathways, potassium channels, intracellular calcium concentration, nitric oxide, heat-shock proteins, as well as neutrophil and platelets adhesion to the endovascular cells appear to be involved in ischemia and reperfusion injury [22,23].

Although the current trial focuses on a sevoflurane intervention after on-pump cardiac surgery (postconditioning), several studies have elucidated the effect of the application of volatile anesthetics during the whole surgical procedure, called conditioning, or preconditioning, when applied before initiation of the ischemic phase. Data from Cromheecke *et al. *[24] reveal that patients randomized to a sevoflurane anesthesia for aortic valve replacement have, in comparison with the propofol group, have a better postoperative cardiac function in the presence of lower troponin I values. Landoni *et al. *[25], however, could not show similar results in patients undergoing mitral valve replacement with a desflurane preconditioning. Bignami and co-workers [26] did not assess a difference in postoperative troponin release in patients with coronary artery disease undergoing mitral valve surgery with a propofol anesthesia throughout the surgical procedure versus a sevoflurane anesthesia before and after CPB (pre- and postconditioning).

The first studies focusing on cardiac postconditioning were performed *in vitro *as well as *in vivo*. Pravdid and his group [27] showed in an ischemia-reperfusion model of cardiomyocytes that isoflurane was protective when applied in reoxygenation. Similarly, in an isolated rat heart model, postconditioning with sevoflurane attenuated infarct size and improved cardiac function [28]. De Hert *et al. *[11] were able to show cardioprotection with sevoflurane postconditioning in patients undergoing CABG surgery. Hellström *et al. *[29] performed a postconditioning trial similar to the current one, sedating patients after CABG surgery with either propofol or sevoflurane for 2 hours only. Results of that trial including patients undergoing CABG surgery with sevoflurane anesthesia and a postoperative randomization to propofol or sevoflurane sedation are comparable to those of the current study with patients after valve replacement: although troponin T values, measured at 12 hours after surgery, were the same in both groups, an attenuated statistically significant increase of troponin T, measured from baseline to peak values, was observed in the sevoflurane arm.

Whether volatile anesthetics are capable of providing organ protection in events of clear ischemia-reperfusion only remains questionable. This may imply myocardial injury after cardiopulmonary bypass, but also with a myocardial ischemia in the perioperative phase of patients with cardiac surgery. This topic was extensively discussed and elegantly summarized in an editorial from De Hert [30]. Another important aspect is the time point of application of volatile anesthetics in relation to the event of ischemia-reperfusion, which should not be underestimated. Volatile anesthetics might induce a protective signaling in the myocardial cells in defined windows only. Such considerations would also explain the results of the randomized controlled study performed by Zangrillo *et al. *[31], in which conditioning with volatile anesthetics in patients undergoing noncardiac surgery was not superior to an anesthesia regimen with propofol. Therefore evidence of the guidelines from the American College of Cardiology/American Heart Association [32] recommending volatile anesthetics for maintenance of general anesthesia in patients at risk for myocardial events is questionable [33].

Whereas the myocardium-specific marker troponin T was significantly lower in the sevoflurane arm on the first postoperative day, no difference could be detected for CK-MB. This could be due to a different kinetic of these markers with a faster peak value for the CK-MB compared with troponin T on injury. Moreover, CK-MB was not recommended for monitoring patients to detect perioperative myocardial infarction after cardiac surgery. Sensitivity and specificity of troponin T seem to be superior [34]. This might explain the observed differences in our trial. Myoglobin values peak within hours after a myocardial lesion and therefore were not detected at the time points of 4 hours and POD1. Additionally, myoglobin is not a reliable parameter for myocardial infarction in general.

This study confirms the already well-known cardioprotective properties of sevoflurane as compared with propofol. Remarkably, and in contrast to all other clinical studies on pharmacologic conditioning with volatile anesthetics, we have used for the first time a late postconditioning protocol exposing patients to low concentrations of sevoflurane only in the postoperative phase and for a relatively short period of 4 hours, whereas patients in the study of Hellström *et al. *[29] were also exposed to sevoflurane during surgery. Application of volatile anesthetics during surgery might reflect a certain bias for the study result. In our trial, patients were exclusively exposed to sevoflurane during the sedation phase in the ICU for a minimum of 4 hours.

An important aspect of our study that must be considered is the fact that only biomarkers were analyzed. If such positive results would claim the potential of being translated into clinical routine, similar findings from additional studies would be necessary. Such trials would require large numbers of patients. Nevertheless, using biomarkers is certainly a reliable approach for a first clinical trial, which allows linking the finding with current preclinical work including *in vitro *and animal models as a first proof of principle.

Propofol is among the most commonly used substances for sedation in the ICU because it is easily administered and does not accumulate as do other substances (for example, benzodiazepines) if used for long-term sedation. However, because the delivery of volatile anesthetics in the ICU has become available with the AnaConDa system, isoflurane and sevoflurane are new options for sedation of postoperative and critically ill patients. It not only allows an alternative method of postoperative sedation, but also might offer new options in selective and delayed use of organ-protective strategies with ischemia-reperfusion injury. Moreover, postconditioning could be adopted as a "therapeutic" option to prevent further cell and organ damage after any kind of injury, such as inflammation or trauma.

Several potential limitations of the study are acknowledged. First, it is known that the controlled substance propofol also has protective characteristics. The antiinflammatory effect of propofol attenuating cytokine response has been demonstrated extensively [35,36]. At least some part of this antiinflammatory effect is also attributed to its containing ethylenediaminetetraacetic acid (EDTA), which is an additive in some of the commercially available propofol formulations [37]. However, these properties would rather have diminished the observed group differences in cardiac injury markers and in postoperative pulmonary complications between sevoflurane and propofol in our trial. From this point of view, the findings may be even more impressive if another substance not having possible antiinflammatory properties had been used for comparison instead of propofol.

Second, the postconditioning phase was relatively short (4 hours) and did not exceed this time interval. In addition, the applied dose of sevoflurane reflecting an age-adjusted 0.5 MAC was relatively low when compared with anesthetic doses during the operation and some supraanesthetic concentrations used in preconditioning settings [38]. In this context, the observed results appear very robust, because higher concentrations of volatile anesthetics could possibly exert even more pronounced organ-protective effects.

Third, as we were interested only in the per-protocol data, we did not perform an intention-to-treat analysis, which would imply a calculation of the data from all randomized patients, but a per-protocol analysis.

Fourth, although chlorofluorocarbons (CFCs) are firmly linked with stratospheric ozone depletion and global atmospheric warming, halogenated anesthetics only contribute for a very small amount of man-made air pollution with these substances [39]. A widespread use of low-dose volatile anesthetics for sedation with low flow rates should reduce the negative impact on the environment and thereby not influence global warming [40].

## Conclusions

The data presented in this investigation suggest that anesthetic postconditioning with sevoflurane might mediate cardiac protection, even with the postoperative application of low-dose sevoflurane. The clinical implementation of these agents can offer an additional tool in the treatment or prevention of ischemic organ dysfunction in the postoperative period. Further studies are warranted with clinical end points focusing on morbidity and mortality.

## Key messages

• Short application of sevoflurane in the early postoperative phase decreases troponin T.

• Sedation with a volatile anesthetic in intensive care units is a possible option to attenuate organ injury.

## Abbreviations

ACC: aortic cross-clamp; ANACONDA: anesthetic-conserving device; BIPAP: biphasic positive airway pressure; BIS: bispectral index; CCS: Canadian Cardiovascular Society; CABG: coronary artery bypass graft; CFC: chlorofluorocarbon; CK: creatine kinase; CK-MB: myocardium-specific creatine kinase; ECC: extracorporeal circulation; EDTA: ethylenediaminetetraacetic acid; FEV_1_: forced expiratory volume in 1 second; FRC: functional residual capacity; ICU: intensive care unit; MAC: minimum alveolar concentration; OLV: one-lung ventilation: PEEP: positive end-expiratory pressure; POD: postoperative day; PONV: postoperative nausea and vomiting.

## Competing interests

The authors have no competing interests. BBS has received a research grant from Abbott AG, Baar, Switzerland, for basic and translational research (Abbott was not involved in the study design, analyzing the data, or in the process of writing the manuscript).

## Authors' contributions

MPS, MS, ERS, TAN, and BBS designed the trial. MPS, WB, MS, DRS, VF, OMT, ERS, and BBS performed the trial. MPS, MS, and TP analyzed the data. MPS, MS, WB, TP, MS, PD, ERS, DS, TAN, and BBS wrote the manuscript. All authors read and approved the final manuscript.

## References

[B1] Riedel BJ Grattan A Martin CB Gal J Shaw AD Royston D Long-term outcome of patients with perioperative myocardial infarction as diagnosed by troponin i after routine surgical coronary artery revascularization J Cardiothorac Vasc Anesth 2006 16 781 787 10.1053/j.jvca.2006.01.015 17138080

[B2] Cremer J Martin M Redl H Bahrami S Abraham C Graeter T Haverich A Schlag G Borst HG Systemic inflammatory response syndrome after cardiac operations Ann Thorac Surg 1996 16 1714 1720 10.1016/0003-4975(96)00055-0 8651772

[B3] Wan S LeClerc JL Vincent JL Inflammatory response to cardiopulmonary bypass: mechanisms involved and possible therapeutic strategies Chest 1997 16 676 692 10.1378/chest.112.3.676 9315800

[B4] Grover FL The Society of Thoracic Surgeons National Database: current status and future directions Ann Thorac Surg 1999 16 367 373 discussion 374-366 10.1016/S0003-4975(99)00599-8 10475399

[B5] Levy JH Tanaka KA Inflammatory response to cardiopulmonary bypass Ann Thorac Surg 2003 16 S715 S720 10.1016/S0003-4975(02)04701-X 12607717

[B6] Meiser A Laubenthal H Inhalational anaesthetics in the ICU: theory and practice of inhalational sedation in the ICU, economics, risk-benefit Best Pract Res Clin Anaesthesiol 2005 16 523 538 10.1016/j.bpa.2005.02.006 16013698

[B7] Kong KL Willatts SM Prys-Roberts C Isoflurane compared with midazolam for sedation in the intensive care unit BMJ 1989 16 1277 1280 10.1136/bmj.298.6683.1277 2500195 PMC1836531

[B8] Meiser A Sirtl C Bellgardt M Lohmann S Garthoff A Kaiser J Hugler P Laubenthal HJ Desflurane compared with propofol for postoperative sedation in the intensive care unit Br J Anaesth 2003 16 273 280 10.1093/bja/aeg059 12594136

[B9] Rohm KD Wolf MW Schollhorn T Schellhaass A Boldt J Piper SN Short-term sevoflurane sedation using the Anaesthetic Conserving Device after cardiothoracic surgery Intensive Care Med 2008 16 1683 1689 10.1007/s00134-008-1157-x 18500419

[B10] Belhomme D Peynet J Louzy M Launay JM Kitakaze M Menasche P Evidence for preconditioning by isoflurane in coronary artery bypass graft surgery Circulation 1999 16 19 Suppl II340 II344 10567326 10.1161/01.cir.100.suppl_2.ii-340

[B11] De Hert SG Van der Linden PJ Cromheecke S Meeus R Nelis A Van Reeth V ten Broecke PW De Blier IG Stockman BA Rodrigus IE Cardioprotective properties of sevoflurane in patients undergoing coronary surgery with cardiopulmonary bypass are related to the modalities of its administration Anesthesiology 2004 16 299 310 10.1097/00000542-200408000-00009 15277911

[B12] Guarracino F Landoni G Tritapepe L Pompei F Leoni A Aletti G Scandroglio AM Maselli D De Luca M Marchetti C Crescenzi G Zangrillo A Myocardial damage prevented by volatile anesthetics: a multicenter randomized controlled study J Cardiothorac Vasc Anesth 2006 16 477 483 10.1053/j.jvca.2006.05.012 16884976

[B13] Tritapepe L Landoni G Guarracino F Pompei F Crivellari M Maselli D De Luca M Fochi O D'Avolio S Bignami E Calabro MG Zangrillo A Cardiac protection by volatile anaesthetics: a multicentre randomized controlled study in patients undergoing coronary artery bypass grafting with cardiopulmonary bypass Eur J Anaesthesiol 2007 16 323 331 10.1017/S0265021506001931 17156509

[B14] Melloni C Anesthesia and sedation outside the operating room: how to prevent risk and maintain good quality Curr Opin Anaesthesiol 2007 16 513 519 10.1097/ACO.0b013e3282f06ba6 17989542

[B15] Marcos-Vidal JM Gonzalez R Garcia C Soria C Out-of-operating room anesthesia: use of the AnaConDa vaporizer with anesthesia J Clin Anesth 2012 16 346 347 10.1016/j.jclinane.2011.05.008 22608592

[B16] Mapleson WW Effect of age on MAC in humans: a meta-analysis Br J Anaesth 1996 16 179 185 10.1093/bja/76.2.179 8777094

[B17] Hausenloy DJ Yellon DM Preconditioning and postconditioning: underlying mechanisms and clinical application Atherosclerosis 2009 16 334 341 10.1016/j.atherosclerosis.2008.10.029 19081095

[B18] Sanada S Komuro I Kitakaze M Pathophysiology of myocardial reperfusion injury: preconditioning, postconditioning and translational aspects of protective measures Am J Physiol Heart Circ Physiol 2011 10.1152/ajpheart.00553.2011 21856909

[B19] De Hert SG ten Broecke PW Mertens E Van Sommeren EW De Blier IG Stockman BA Rodrigus IE Sevoflurane but not propofol preserves myocardial function in coronary surgery patients Anesthesiology 2002 16 42 49 10.1097/00000542-200207000-00007 12131102

[B20] Julier K da Silva R Garcia C Bestmann L Frascarolo P Zollinger A Chassot PG Schmid ER Turina MI von Segesser LK Pasch T Spahn DR Zaugg M Preconditioning by sevoflurane decreases biochemical markers for myocardial and renal dysfunction in coronary artery bypass graft surgery: a double-blinded, placebo-controlled, multicenter study Anesthesiology 2003 16 1315 1327 10.1097/00000542-200306000-00004 12766638

[B21] Landoni G Biondi-Zoccai GG Zangrillo A Bignami E D'Avolio S Marchetti C Calabro MG Fochi O Guarracino F Tritapepe L De Hert S Torry G Desflurane and sevoflurane in cardiac surgery: a meta-analysis of randomized clinical trials J Cardiothorac Vasc Anesth 2007 16 502 511 10.1053/j.jvca.2007.02.013 17678775

[B22] Zaugg M Schaub MC Signaling and cellular mechanisms in cardiac protection by ischemic and pharmacological preconditioning J Muscle Res Cell Motil 2003 16 219 249 10.1023/A:1026021430091 14609033

[B23] Pagel PS Postconditioning by volatile anesthetics: salvaging ischemic myocardium at reperfusion by activation of prosurvival signaling J Cardiothorac Vasc Anesth 2008 16 753 765 10.1053/j.jvca.2008.03.005 18922439

[B24] Cromheecke S Pepermans V Hendrickx E Lorsomradee S Ten Broecke PW Stockman BA Rodrigus IE De Hert SG Cardioprotective properties of sevoflurane in patients undergoing aortic valve replacement with cardiopulmonary bypass Anesth Analg 2006 16 289 296 10.1213/01.ane.0000226097.22384.f4 16861404

[B25] Landoni G Calabro MG Marchetti C Bignami E Scandroglio AM Dedola E De Luca M Tritapepe L Crescenzi G Zangrillo A Desflurane versus propofol in patients undergoing mitral valve surgery J Cardiothorac Vasc Anesth 2007 16 672 677 10.1053/j.jvca.2006.11.017 17905272

[B26] Bignami F Pilotti E Bertoncelli L Ronzi P Gulli M Marmiroli N Magnani G Pinti M Lopalco L Mussini C Ruotolo R Galli M Cossarizza A Carsoli C Stable changes in CD4+ T-lymphocyte microRNA expression following exposure to HIV-1 Blood 2012 10.1182/blood-2011-09-379503 22286198

[B27] Pravdic D Mio Y Sedlic F Pratt PF Warltier DC Bosnjak ZJ Bienengraeber M Isoflurane protects cardiomyocytes and mitochondria by immediate and cytosol-independent action at reperfusion Br J Pharmacol 2010 16 220 232 10.1111/j.1476-5381.2010.00698.x 20423337 PMC2874845

[B28] Fang NX Yao YT Shi CX Li LH Attenuation of ischemia-reperfusion injury by sevoflurane postconditioning involves protein kinase B and glycogen synthase kinase 3 beta activation in isolated rat hearts Mol Biol Rep 2010 16 3763 3769 10.1007/s11033-010-0030-5 20217242

[B29] Hellstrom J Owall A Bergstrom J Sackey PV Cardiac outcome after sevoflurane versus propofol sedation following coronary bypass surgery: a pilot study Acta Anaesthesiol Scand 2011 16 460 467 10.1111/j.1399-6576.2011.02405.x 21342154

[B30] De Hert SG Cardioprotection by volatile anesthetics: what about noncardiac surgery? J Cardiothorac Vasc Anesth 2011 16 899 901 10.1053/j.jvca.2011.08.004 21955826

[B31] Zangrillo A Testa V Aldrovandi V Tuoro A Casiraghi G Cavenago F Messina M Bignami E Landoni G Volatile agents for cardiac protection in noncardiac surgery: a randomized controlled study J Cardiothorac Vasc Anesth 2011 16 902 907 10.1053/j.jvca.2011.06.016 21872490

[B32] Fleisher LA Beckman JA Brown KA Calkins H Chaikof EL Fleischmann KE Freeman WK Froehlich JB Kasper EK Kersten JR Riegel B Robb JF ACC/AHA 2007 guidelines on perioperative cardiovascular evaluation and care for noncardiac surgery: a report of the American College of Cardiology/American Heart Association Task Force on Practice Guidelines (Writing Committee to Revise the 2002 Guidelines on Perioperative Cardiovascular Evaluation for Noncardiac Surgery) developed in collaboration with the American Society of Echocardiography, American Society of Nuclear Cardiology, Heart Rhythm Society, Society of Cardiovascular Anesthesiologists, Society for Cardiovascular Angiography and Interventions, Society for Vascular Medicine and Biology, and Society for Vascular Surgery J Am Coll Cardiol 2007 16 e159 e241 10.1016/j.jacc.2007.09.003 17950140

[B33] Landoni G Fochi O Zangrillo A Cardioprotection by volatile anesthetics in noncardiac surgery? No, not yet at least J Am Coll Cardiol 2008 16 1321 1322 1321; author reply 18371566 10.1016/j.jacc.2007.12.020

[B34] Januzzi JL Lewandrowski K MacGillivray TE Newell JB Kathiresan S Servoss SJ Lee-Lewandrowski E A comparison of cardiac troponin T and creatine kinase-MB for patient evaluation after cardiac surgery J Am Coll Cardiol 2002 16 1518 1523 10.1016/S0735-1097(02)01789-8 11985917

[B35] Taniguchi T Yamamoto K Ohmoto N Ohta K Kobayashi T Effects of propofol on hemodynamic and inflammatory responses to endotoxemia in rats Crit Care Med 2000 16 1101 1106 10.1097/00003246-200004000-00032 10809290

[B36] Taniguchi T Kanakura H Yamamoto K Effects of posttreatment with propofol on mortality and cytokine responses to endotoxin-induced shock in rats Crit Care Med 2002 16 904 907 10.1097/00003246-200204000-00032 11940767

[B37] Marik PE Propofol: an immunomodulating agent Pharmacotherapy 2005 16 5 Pt 2 28S 33S 15899746 10.1592/phco.2005.25.5_part_2.28s

[B38] Beck-Schimmer B Breitenstein S Urech S De Conno E Wittlinger M Puhan M Jochum W Spahn DR Graf R Clavien PA A randomized controlled trial on pharmacological preconditioning in liver surgery using a volatile anesthetic Ann Surg 2008 16 909 918 10.1097/SLA.0b013e31818f3dda 19092335

[B39] Langbein T Sonntag H Trapp D Hoffmann A Malms W Roth EP Mors V Zellner R Volatile anaesthetics and the atmosphere: atmospheric lifetimes and atmospheric effects of halothane, enflurane, isoflurane, desflurane and sevoflurane Br J Anaesth 1999 16 66 73 10.1093/bja/82.1.66 10325839

[B40] Ryan SM Nielsen CJ Global warming potential of inhaled anesthetics: application to clinical use Anesth Analg 2010 16 92 98 20519425 10.1213/ANE.0b013e3181e058d7

